# The viral origins of breast cancer

**DOI:** 10.1186/s13027-024-00595-2

**Published:** 2024-08-26

**Authors:** James S. Lawson, Wendy K. Glenn

**Affiliations:** University of New South Wales, Sydney, Australia

## Abstract

During the past two decades evidence has been developed that indicates a handful of viruses with known oncogenic capacity, have potential roles in breast cancer. These viruses are mouse mammary tumour virus (MMTV - the cause of breast cancer in mice), high-risk human papilloma viruses (HPV-the cause of cervical cancer), Epstein Barr virus (EBV-the cause of lymphomas and naso-pharyngeal cancer) and bovine leukemia virus (BLV - the cause of cancers in cattle). These viruses may act alone or in combination. Each of these viruses are significantly more prevalent in breast cancers than in normal and benign breast tissue controls. The odds ratios for the prevalence of these viruses in breast cancer compared to normal and benign breast controls, are based on case control studies - MMTV 13·40, HPV 5.56, EBV 4·43 and BLV 2·57. The odds ratios for MMTV are much greater compared to the other three viruses. The evidence for a causal role for mouse mammary tumour virus and high risk for cancer human papilloma viruses in human breast cancer is increasingly comprehensive. The evidence for Epstein Barr virus and bovine leukemia virus is more limited. Overall the evidence is substantial in support of a viral cause of breast cancer.

## Introduction

Viruses have long been suspected as having a causal role in breast cancer but the evidence has not been sufficient to establish causation. However, during the past two decades new evidence has been developed that indicates a handful of viruses with known oncogenic capacity, are the probable underlying causes of initiating breast cancer. These viruses are mouse mammary tumour virus (MMTV - the cause of breast cancer in mice), high-risk human papilloma viruses (HPV-the cause of cervical cancer), Epstein Barr virus (EBV-the cause of lymphomas and naso-pharyngeal cancer) and bovine leukemia virus (BLV - the cause of cancers in cattle). These viruses may act alone or in combination. Each of these viruses are significantly more prevalent in breast cancers than in normal and benign breast tissue controls.

95% of breast cancers are sporadic and historically have no known cause. Risks as compared to causal factors for breast cancer include familial susceptibility, early menarche, late menopause and late age first pregnancy. Radiation is an established risk factor. Genetics plays a small but important role in breast cancer. Inherited mutations in BRCA1 and 2 genes lead to an increased risk of 3 to 5% of breast cancer.

The evidence indicating a causal role for mouse mammary tumour virus is comprehensive but less so for the other viruses. In addition the odds ratios for MMTV are much greater compared to the other three viruses. There has been intense research interest in MMTV for over 90 years. For this reason there is more detailed information about MMTV in this review compared to the more recent studies of other viruses. However the role of high risk for cancer HPVs in breast cancer is also important and will be outlined in detail.

The history of research into MMTV and breast cancer has been published by Generoso Bevilacqua of Pisa, Italy [1]. The role of MMTV in breast cancer has been the dominant research interest. MMTV also appears to have a role in biliary cholangitis and autoimmune liver disease and has been identified in ovarian, prostate, endometrial and skin cancers [2–4].

The odds ratios (the ratio of the odds of the event in an exposed group versus a non-exposed group) for the prevalence of these viruses in breast cancer compared to normal and benign breast controls, are based on case control studies - MMTV 13·40, HPV 5.56, EBV 4·43 and BLV 2.57 [5–8].

## Search strategy and selection criteria

PubMed Central is the main source of publications in this review. References listed in published articles have also been searched. We have used an extended version of the classic A.Bradford Hill causal criteria to assess the evidence concerning the role of these oncogenic viruses [9]. The extended Hill causal criteria include – identification of the causal pathogen, strength of association between the pathogen and the cancer, consistency, temporality (timing), experiment, analogy, means of transmission and oncogenic mechanisms.

## Meta-analyses of case control studies of oncogenic viruses and breast cancer

MMTV, HPVs, EBV and BLV are significantly and consistently more prevalent in breast cancers than in normal and benign breast controls. These data are shown as odds ratios in Table 1 based on meta-analyses of case - control studies. Since the publication of these meta-analyses additional case control studies have been published for each of the four oncogenic viruses. These publications have been added to the tables.

**Table 1:** Meta-analyses of virus positive breast cancers compared to normal / benign breast controls

Author	Number of studies	Breast cancer Virus +/cases	Normal / benign breast controls Virus +/cases	Odds ratios (Confidence intervals)
Wang 2021 [5] MMTV	28	1287/4015 32%	24/999 2.4%	13·40 (11·44–15·36)
Awan 2023 [6] HPV	45	1145/4355 26.3%	163/2361 6.9%	5.56 (3.67–8.41)
Agolli 2023 [7] EBV	24	555/1989 28%	83/1034 8%	4·43 (3.47–5.66)
Khatami 2020 [8] BLV	9	334/826 40%	215/898 24%	2·57 (1·45–4·56)

## Mouse mammary tumour virus (MMTV)

Mouse mammary tumour virus (MMTV) is a retrovirus. In 1936, John Bittner discovered a pathogenic agent, which could be transmitted by milk from mouse mothers with breast cancer to their pups who as adults, later developed breast cancer [10]. Later, Samuel Graff and colleagues identified viral particles in mouse milk that could cause mammary cancer when intraperitoneally injected into laboratory strains of mice [11]. These retroviral RNA particles became known as mouse mammary tumour virus and are strongly linked with breast cancer in mice [12].

**Fig. 1:**
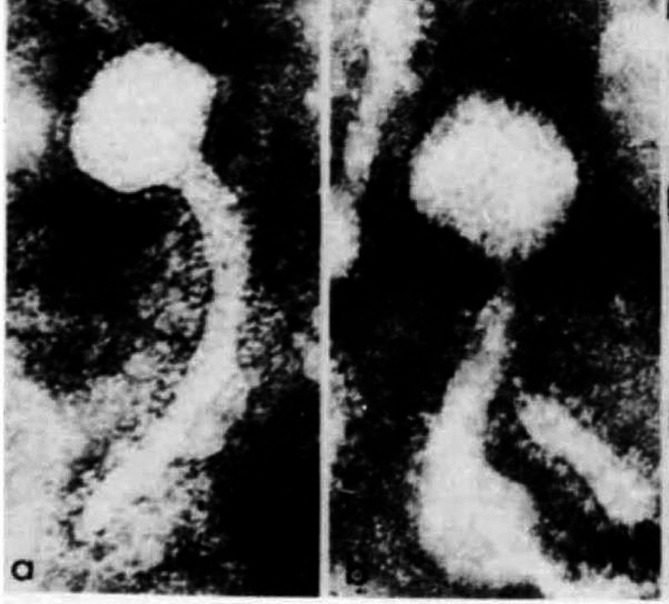
MMTV particles identified in (**a**) human milk, (**b**) mouse milk (×18,000) by electron microscopy. Note the long curved tail [12]. (Republished with permission from Nature)

In 1971, Moore et al. using electron microscopy showed human milk containing viral particles with morphology identical to mouse mammary tumour virus in mouse milk [13] (Fig. 1). The images show an unusual shape for a virus including a long tail. This shape is probably because MMTVs begin as type A particles, later they become type B particles via a process of “budding” on the virus surface which takes place due to sophisticated molecular events [14].

The nucleotide sequences and structure of MMTV-like viral sequences identified in human breast cancer tissues are virtually identical to the MMTV sequences identified in mouse mammary tumours [15]. The same 63 cancer-related genes have been identified in both human and mouse breast cancers [16]. The MMTV envelope and capsid protein expression is similar in both mouse and human breast cancers [17]. MMTV-associated tumour histology is similar in both mouse and human breast cancers [18]. In MMTV associated mouse breast cancers, the oncogene Wnt-1 is highly expressed. In humans, the influence of MMTV on human breast cells leads to abnormally high Wnt-1 expression [19].

In 1972 using molecular hybridisation Axel et al. demonstrated that MMTV was present in 62% of 29 human invasive breast cancers compared with 0% in benign and normal breast tissue controls [20]. In 1987, Moore et al. identified the complete nucleotide sequence of the mouse mammary tumour virus [21]. In 1981 Day et al. identified increased levels of the antibody to MMTV in 18.6% of US women with breast cancer compared to 2.8% of healthy women [22].

In 1995 using PCR, the Pogo group from New York identified the MMTV envelope gene in 38.5% of 314 human breast cancer specimens compared to 7% of 29 benign human breast specimens and in one of 27 normal human breast specimens [23]. These findings were confirmed by other research groups [24, 25]. The MMTV envelope and other cancer related gene sequences are identical in both human breast cancers and mouse mammary tumours [16, 26]. Accordingly, it is likely that MMTV is the same virus in both human and mouse breast cancers.

### Strength and consistency of association between MMTV and human breast cancer

#### Case control studies

There are 26 case control studies in which MMTV has been identified. MMTV was not identified in 12 additional studies. The studies in which MMTV was identified, show a consistent pattern of outcomes, namely, positive identification of MMTV in 10 to 70% of breast cancers and zero to less than 5% in benign and normal breast tissue controls. In Western women, the prevalence of MMTV in breast cancers is approximately 30 to 40%. In China and Korea, the prevalence of MMTV is 10 to 20%. Positive MMTV case control studies are shown in Table 2. Negative MMTV breast cancer studies are shown in Table 3.

The implication is that there is a strong association between MMTV and human breast cancer.

There appear to be two reasons for the negative outcomes in 12 case control studies. One, MMTV may not be present in some human and mouse populations (see the epidemiology section below), and second the use of inadequate PCR techniques. This problem has been highlighted by Pogo et al. who demonstrated that the techniques used by Park et al. gave negative outcomes as compared to positive outcomes using alternative techniques [27, 28]. A further difficulty is the low MMTV viral load. Using quantitative PCR Mazzanti et al. (2011) demonstrated there is a dramatic decrease in MMTV viral load as the cancer progresses from ductal carcinoma in situ to invasive carcinoma [29]. They demonstrated there can be a complete loss of the virus in invasive ductal carcinoma.

**Table 2:** MMTV breast cancer case control studies. S = significant. Ns = not significant

Study	Country	Method	Breast cancer	Breast control	Significance
Axel 1972 [20]	US	Molecular hybridisation	19/29 66%	0/15 0%	0.004 s
Mesa-Tajada 1978 [30]	US	IHC	15/131 39%	0/18 0%	0.001s
Wang 1995 [23]	US	PCR	121/314 38.5%	2/107 2%	0.001s
Etkind 2000 [31]	US	PCR	27/73 37%	0/35 0%	0.001s
Melana 2001 [32]	US	PCR	32/106 30%	1/106 1%	0.001s
Melana 2002 [33]	Argentina	PCR	23/74 31%	1/10 10%	0.003s
Ford 2003 [24]	Australia	PCR	19/45 42%	2/111 2%	0.001s
Ford 2004 [34]	Australia	PCR	45/144 31%	0/111 0%	0.001s
Zammarchi 2006 [25]	Italy	PCR	15/45 33%	0/8 0%	0.008 s
Hachana 2008 [35]	Tunisia	PCR	17/122 14%	0/122 0%	0.001s
Lawson 2010 [36]	Australia	In situ PCR	33/74 45%	0/29 0%	0.001s
Mazzanti 2011 [29]	Italy	PCR	47/69 68%	0/20 0%	0.001s
Glenn 2012 [37]	Australia	PCR	39/50 78%	13/40 33%	0.045 s
Slaoui 2014 [38]	Morocco	PCR	24/42 57%	6/18 33%	0.312 ns
Cedro-Tanda 2014 [39]	Mexico	PCR	57/458 12%	72/458 16%	0.308 ns
Naushad 2014 [40]	Pakistan	PCR	83/250 29%	0/15 0%	0.001s
Reza 2015 [41]	Iran	PCR	12/100 12%	0/100 0%	0.001s
Shariatpanahi 2017 [42]	Iran	PCR	19/59 32%	3/59 5%	0.002 s
Al Dossary 2018 [43]	Saudi Arabia	PCR	6/101 6%	0/51 0%	0.082 ns
Seo 2019 [44]	Korea	PCR	12/128 9%	0/128 0%	0.013 s
Al Hamad 2020 [45]	Jordan	PCR	11/100 11%	0/20 0%	0.023 s
Loutfy 2021 [46]	Egypt	PCR	38/50 76%	0/10 0%	0.001s
Wang 2021 [5]	China	PCR	21/119 18%	2/50 4%	0.05 s
Khalid 2021 [47]	Pakistan	PCR	69/105 66%	2/15 13%	0.023 s
Gupta 2021 [48]	Croatia	PCR	5/70 7%	0/16 0%	0.056 ns
Gupta 2022 [49]	Qatar	PCR	11/74 15%	0/14 0%	0.001s

**Table 3:** MMTV breast cancer – negative results

Study	Country	Method	Negative MMTV breast cancer
Zangen 2002 [50]	Italy	PCR	0/18
Witt 2003 [51]	Austria	PCR	0/50
Mant 2004 [52]	United Kingdom	PCR	0/44
Bindra 2007 [53]	Sweden	PCR	0/18
Frank 2008 [54]	Germany	Hybridisation	0/23
Fukuoka 2008 [55]	Japan	PCR/hybridisation	0/46
Park 2011 [28]	Australia	PCR	0/42
Motamedifar 2012 [56]	Iran	PCR	0/50
Tabriz 2013 [57]	Iran	PCR	0/40
Morales-Sanchez 2013 [58]	Mexico	PCR	0/65
Perzova 2017 [59]	USA	PCR	0/9
Fekete 2023 [60]	Romania	PCR	0/75

#### Contamination issues

It has been argued that some MMTV positive human breast cancer results may be due to contamination with mouse DNA. Both Mazzanti et al. and Nartey et al. excluded mouse DNA contamination by performing murine mitochondrial DNA and intracisternal A-particle long terminal repeats by PCR [61, 62]. In addition MMTV has been independently identified in breast cancers in over 22 countries. It is most unlikely that contamination occurred in each of these laboratories.

#### MMTV and human breast cancer histology

Some human breast cancer specimens, in which MMTV-like env DNA sequences have been identified, were shown to have histological characteristics (morphology) similar to MMTV-associated mouse mammary tumours [18]. These observations are compatible with an association between the presence of MMTV-like env DNA sequences and some human breast cancers.

#### Epidemiology

The prevalence of breast cancer varies greatly between countries [63]. The US, Australia, the Netherlands, France and Germany have high rates - over 95 cases per 100,000 age-adjusted females. Japan, China, and India have low rates—between 30 and 45 cases per 100,000 age-adjusted females. There are marked differences in the prevalence of breast cancer between Western and Eastern Europe. In the United Kingdom, the Netherlands, France and Italy, the prevalence of breast cancer is over 95 as compared to Russia and the Baltic countries, with a prevalence of less than 50 per 100,000 age-adjusted females. There are also differences in parts of northern as compared to southern China [5].

While there are several reasons including food consumption patterns, which may account for the differences in the prevalence of breast cancer, an additional reason is the distribution of MMTV-infected mice [64, 65]. *Mus domesticus*, which is the common house mouse in Western countries, carries more infectious MMTV than does *Mus musculus* [64]. *Mus domesticus* mice are mainly located in Western Europe and *Mus musculus* in Eastern Europe. *Mus domesticus* is more prevalent in northern as compared to southern China [5]. The prevalence of MMTV positive human breast cancer is significantly higher in Western as compared to eastern Europe and in northern China (23%) as compared to southern China. (6%). In a meta – analysis Wang et al. also showed there was a significant correlation between the prevalence of MMTV positive breast cancer in different countries and the global distribution of *Mus domesticus* [5]. Recent developments provide support for this hypothesis [65]. *Mus domesticus* mouse population outbreaks in Australia and New Zealand are associated with a modest increase in breast cancer incidence rates with a lag of approximately 3 years [65].

#### Serology studies

The prevalence of MMTV antibodies in the serum of women with breast cancer is consistently five-fold higher than in the sera of women with benign breast conditions or in normal women (Table 4). An exception is the study by Goerdert et al. (2006) who did not identify any MMTV antibodies in the sera of 92 women with breast cancer (however, MMTV gp52 was not identified in the positive controls) [66]. Zhang et al. more recently used ELISA gp 52 to demonstrate MMTV antibodies in 10% of sera from women with breast cancer compared to 2% of controls (*p* = 0.017) [67].

**Table 4:** Mouse mammary tumour virus breast cancer serology

Study	Country	Breast cancer	Controls	Significance
Muller 1972 [68]	Germany	75/228 33%	11/95 12%	0.002 s
Ogawa 1978 [69]	Japan	26/43 60%	4/37 11%	0.001s
Mehta 1978 [70]	India	26/34 76%	0/10 0%	0.001s
Witkin 1979 [71]	US	11/65 17%	2/60 3%	0.001 s
Imai 1979 [72]	Japan	49/89 55%	18/68 27%	0.020 s
Witkin 1980 [73]	US	14/54 26%	5/63 8%	0.026s
Day 1981 [22]	US	27/145 19%	1/36 3%	0.026 s
Nagayoshi 1981 [74]	Japan	34/96 36%	3/59 5%	0.026 s
Tomana 1981 [75]	US	56/137 41%	2/56 4%	0.001 s
Zotter 1981 [76]	Germany	84/367 23%	11/184 6%	0.001 s
Holder 1983 [77]	US	41/52 79%	2/18 11%	0.004 s
Litvinov 1984 [78]	Russia	51/92 55%	3/94 3%	0.001 s
Chattopadhyah 1984 [79]	India	14/14 100%	0/13 0%	0.004 s
Kovarik 1989 [80]	Czech	2/60 3%	0/60 0%	0.226 ns
Goerdert 2006 [66]	US	0/92		
Zhang 2020 [67]	Canada	10/98 10%	2/98 2%	0.017 s

#### Genetics

The Bevilacqua group in Pisa, Italy, have shown that MMTV-like sequences are not significantly associated with hereditary breast carcinoma [81]. 30% of 56 sporadic breast cancers contain MMTV sequences compared to 4% of 47 BRCA hereditary breast cancers (*p* < 0.001) [81].

#### Temporality (timing) of the Association

MMTV has been identified in normal and benign human breast tissues up to 10 years prior to the development of MMTV positive human breast cancers in the same patient [62]. This is an important causal criteria. MMTV has also been identified in the sputum of children [61]. The implication is that breast cancer may take many decades to develop following infections with MMTV. On the other hand Stewart and Chen (2022) have hypothesised that MMTV positive breast cancer can develop within 3 years following mouse epidemics [65]. There is no obvious explanation for these differences.

#### MMTV transmission

Human saliva is a likely means of transmission of MMTV between humans [61]. MMTV gene sequences are present in saliva in 27% of normal children, 11% of normal adults and 57% of women with breast cancer [61]. MMTV gene sequences have been identified in human parotid glands - the source of saliva [61]. Humans have well developed lymphatic structures in the mouth and nose which are possible entry points for MMTV. That MMTV can infect adult mice via nasal lymphoid tissue has been demonstrated experimentally [82]. Accordingly, MMTV can enter mammals, including humans, from a range of sites.

Because MMTV is transmitted via milk from mouse mothers to their pups, the presence of MMTV in human milk has been investigated. MMTV has been identified in 5% of breast milk samples from healthy lactating Australian women [83]. In addition it has been shown that the prevalence of MMTV in human milk is significantly higher among women who are at greater than normal risk of breast cancer [84]. However MMTV transmission via human milk is unlikely to be an influential means of transmission in humans. There are two reasons. One, is the destructive effect of human milk. This was shown experimentally by Sarkar et al. (1973) [85]. Two, because epidemiological studies have not shown associations between breast feeding and breast cancer [86].

MMTV has been identified in mammary tumours in dogs and cats [87, 88]. It is possible, but not proven, that transmission could occur between cats, dogs and humans, as has been shown between mice and humans.

In many countries it is permissible for 1% in weight of cereals to contain mouse or rat faecal material. US regulatory food standards allow up to two pellets of rodent excreta per pint of wheat (US pint = 551 cm^3^) [89]. Because MMTV is endemic in many rodent populations, transmission via rodent faecal material by consumption of uncooked cereals and other foods is possible.

#### Experimental evidence

MMTVs can infect human breast cells in culture and can randomly integrate into the human genome [90]. MMTV proteins have been shown to be capable of malignantly transforming normal human breast epithelial cells [91].

#### Analogy

The life cycle of MMTV is similar in mice and humans. MMTV infects T and B lymphocytes in the Peyer’s lymphocyte patches. MMTV is activated by super antigens which circulate in lymphocytes and enters breast epithelial cells, where they integrate into the mouse and human genome [92].

#### Oncogenic mechanisms

The underlying causal mechanisms by which MMTV may cause human breast cancer are far from clear. MMTV proteins are capable of malignant transformation of normal human breast epithelial cells [91]. An additional mechanism involves the APOBEC enzyme family. APOBEC3B is an enzyme which inhibits retrovirus replication. In humans, inactivating mutations and deletions in APOBEC3B appear to play a role in breast cancer development [93, 94]. Human papilloma viruses have been shown to alter the expression of APOBEC3B, which may reduce its protective effects against MMTV [95].

### Conclusion

The evidence meets the extended Hill causal criteria. A causal role for MMTV-like viruses in human breast cancer is probable.

## Human papilloma virus

High risk for cancer human papilloma viruses (HPV) in breast cancer were first identified in 1992 by Anna Di Lonardo and colleagues working in Rome [96]. Their findings were later confirmed in case control studies all of which demonstrated that the prevalence of high- risk HPVs was consistently higher in breast cancer than in normal and benign breast controls [7].

High-risk HPVs have been identified in breast cancer in 20 countries [6]. High risk HPV types 16 and 18 are predominant in Western women. HPV types 16, 18, 33, 52 and 58 are common in Chinese, Korean, Japanese and Qatari women. In studies in which high-risk HPVs were identified, there were no correlations with breast cancer grade, survival, or steroid receptor expression [97]. The reason for the variations in outcomes is partly due to the difficulty of identifying the extremely low HPV viral loads in breast cancer as compared to cervical cancer [98].

### Strength and consistency of association between HPVs and breast cancer

There have been 46 case control studies in which HPVs have been identified [6]. The prevalence of high risk for cancer HPVs was 1335 (31%) of breast cancers as compared to 163 (9%) of 1838 normal and benign breast tissue controls (*p* = 0.001). A meta-analysis of these studies indicated an odds ratio of 5.56 (*p* = 0.01) for HPV positive breast cancer cases compared to HPV positive controls [6] [Awan 2023]. The outcomes of these case control studies are consistent. These case control studies are shown in Table 5.

**Table 5:** Identification of high risk for cancer human papilloma virus in breast cancers and controls (case control studies)

Study	Country	HPV Breast cancer	HPV Non cancer breast controls	Main HPV types	Significance
Yu 2000 [99]	Japan / China	18/52 35%	0/15 0%	18,33	0.001s
Ren 2003 [100]	China	45/80 56%	2/30 7%	16,18	0.002s
Damin 2004 [101]	Brazil	25/101 25%	0/41 0%	16,18	0.001s
Tsai 2007 [102]	Taiwan	8/62 13%	2/32 6%		0.004s
Choi 2007 [103]	Korea	8/123 7%	0/31 0%	16, 18,58	0.001s
Gumus 2006 [104]	Turkey	37/50 74%	16/50 32%	18,33	0.162 ns
Fan 2008 [105]	China	23/52 44%	1/16 6%	16	0.001s
He 2009 [106]	China	24/40 60%	1/20 5%	16	0.001s
De Leon 2009 [107]	Mexico	15/41 37%	0/43 0%	16,18	0.001s
Mendizabal 2009 [108]	Mexico	3/67 4%	0/40 0%	16,18,33	0.157ns
Heng 2009 [109]	Australia	8/26 31%	3/28 11%	16,18	0.611ns
Mou 2011 [110]	China	4/62 6%	0/46 0%	16,18	0.025s
Sigaroodi 2012 [111]	Iran	15/58 26%	1/41 2%	16,18	0.002s
Frega 2012 [112]	Italy	9/31 29%	0/12 0%	16,18	0.005s
Divani 2012 [113]	Greece	6/35 17%	0/35 0%	16,18	0.025s
Glenn 2012 [37]	Australia	25/50 50%	8/40 20%	16,18	0.006s
Liang 2013 [114]	China	48/224 21%	6/37 16%	16,18,33,58	0.001s
Ahangar2014 [115]	Iran	22/65 34%	0/65 0%	16	0.001s
Ali 2014 [116]	Iraq	60/129 47%	3/41 7%	16,18,33	0.001s
Hong 2014 [117]	China	23/45 51%	1/20 5%	16,18	0.001s
Manzouri 2014 [118]	Iran	10/55 18%	7/51 14%	16	0.083 ns
Fu 2015 [119]	China	25/169 15%	1/83 1%	58	0.001s
Li 2015 [120]	China	3/187 2%	0/92 0%	6,16,18	0.157 ns
Gannon 2015 [121]	Australia	13/78 17%	1/10 10%		0.002s
Lawson 2015 [97]	Australia	27/40 66%	6/21 29%	16,18,58	0.001s
Doosti 2016 [122]	Iran	20/87 23%	0/84 0%	16,18	0.001s
Wang 2016 [123]	China	52/146 36%	3/83 3.6%	16,18,58	0.001s
Zhang 2016 [124]	China	34/325 11%	4/100 4%	16,18	0.001s
Delgardo 2017 [125]	Spain	130/251 52%	49/186 26%	16	0.001s
Ladera 2017 [126]	Venezuela	14/22 64%	1/22 4.5%	16,18,52,56	0.001s
Naushad 2017 [127]	Pakistan	45/250 18%	0/15 0%		0.001s
Islam 2017 [128]	India	203/313 65%	2/21 10%	16,18,33	0.001s
Salman 2017 [129]	United Kingdom	35/74 47%	11/36 18%	16,18,35,45,59	0.001s
Malekpour 2018 [130]	Iran	8/98 8%	0/40 0%	16,18	0.008s
ElAmrani 2018 [131]	Morocco	19/76 25%	1/12 8%	51,52,58	0.001s
Cavalcante 2018 [132]	Brazil	51/103 50%	15/95 16%	6,11,18,31	0.001s
Khodabandehlou 2019 [133]	Iran	35/72 48.6%	5/3116.1%	18	0.003s
Al Hamad 2020 [45]	Jordan	21/100 21%	0/20 0%	16, 18	0.007s
Mofrad 2021 [134]	Iran	7/59 12%	0/11 0%	18	0.004s
El-Seik 2021 [135]	Egypt	16/72 22.2%	0/15 0%	16,18	0.003s
Golrokh 2021 [136]	Iran	7/59 12%	0/15 0%	6,18	0.014s
Guo 2021 [137]	US	25/56 45%	3/9 33%	11, 18	0.005s
Nagi 2021 [138]	Lebanon	66/102 67%	5/14 36%	16,35,45,52,58	0.001s
Alinezhadi 2022 [139]	Iran	7/8 88%	2/9 22%	16	0.025s
Tavakolian 2023 [140]	Iran	9/40 23%	3/ 50 6%	18	0.014s
Khasawneh 2024 [141]	Jordan	27/110 25%	0/30 0%	16,18	0.001s

The prevalence of high risk HPV is consistently higher in all studies of breast cancers as compared to controls

s = significant at 0.05 level ns = not significant at 0.05 level

In a prospective cohort study involving 61,872 subjects in Taiwan, patients with HPV were 1.4 times more likely to develop breast cancer than patients of the same age without HPV [142].

Women who develop HPV associated cervical cancer have a higher risk of developing HPV associated breast cancer [143]. These women are on average 10 years younger at the age of developing breast cancer than older women who develop breast cancer. Younger women are more sexually active and at greater risk of sexually transmitted HPV infections.

#### Oncogenic mechanisms

High-risk HPVs encode proteins, several of which have an oncogenic capacity. The expression of HPV E6 and E7 proteins leads to malignant changes in normal epithelial cells. HPV E6 proteins degrade p53 (a cancer suppressing gene). HPV E7 enhances viral replication and malignant transformation from normal to cancer cells by upregulating Cox-2 which increases inflammation. HPV associated koilocytes have been identified in breast cancers [144]. Koilocytes are large squamous cells with acentric nuclei surrounded by a halo. Koilocytes are the basis of cervical Pap smears. HPV E5 and E6 act early in transformation and lead to the formation of koilocytes. Although several of these HPV oncogenic mechanisms in cervical cancer appear to be involved in breast cancer, additional mechanisms are involved. These may involve the antiviral enzyme APOBEC3B. HPV infections upregulate and lead to mutations in APOBEC3B which increase the risk of breast cancer [95]. Experimental evidence shows that exposure to HPV E6 and E7 proteins can transform and immortalise normal human breast epithelial cells [96]. High risk HPVs of the same type have been identified in benign breast tissues 1 to 11 years before the development of HPV positive breast cancers in the same women [145].

#### Transmission

Sexual intercourse is regarded as the primary route of human papillomavirus (HPV) transmission. However, HPVs are stable viruses which can stay on tissue and other surfaces for many days. There is evidence which indicates HPVs can also be transmitted by non- sexual means from mother to child by fomites, from health care workers to patients and in an unknown manner to adolescent girls with no sexual experience [146]. There is also evidence that HPVs may be transmitted via saliva and blood and from the cervix to the breast by circulating extra cellular vesicles – also known as exosomes [147].

### Conclusions

High risk for cancer HPVs have been consistently identified and are significantly more prevalent in breast cancers than normal and benign breast cancers. In conclusion it is likely that HPVs have a causal role in breast cancer.

## Epstein Barr virus (EBV)

In 1957 surgeon Dennis Burkitt, working in Uganda, identified acute malignant lymphomas in children [148]. Anthony Epstein in collaboration with Burkitt, Bert Achong and Yvonne Barr, identified viral particles in the lymphoma specimens [149]. They later identified the particles as human herpes virus 4 (Epstein Barr virus – EBV). This pioneering work was of universal value because it was the first demonstration that viruses could cause cancer in humans.

In 1995, working in London, Louise Labreque and colleagues made the first identification of EBV in breast cancer [150]. Various EBV genes have since been identified in breast cancer in a wide range of countries.

### Strength and consistency of association between EBVs and breast cancer

EBV positive lymphocytes commonly infiltrate breast cancer cells. Studies which do not identify breast cancer cells separately from infiltrating lymphocytes are not valid. Therefore a careful assessment of each study is required before inclusion in meta-analyses. In a meta-analysis of 24 case control studies by Agolli et al. 2023 the odds ratio of EBV positive breast cancer compared to normal and benign breast controls was 4.43 (*p* = 0.01) [7]. The evidence is consistent that EBVs are significantly more prevalent in breast cancers than controls. These case control studies are shown in Table 6.

**Table 6:** Case control studies Epstein Barr virus and breast cancer

Study	Country	Identification method	EBV positive breast cancer	EBV positive breast controls	Significance
Labreque 1995 [150]	United Kingdom	PCR, ISH	19/91 21%	0/21 0%	0.001
Luqmani 1995 [151]	United Kingdom	PCR, IHC	15/28 54%	0/12 0%	0.001
Bonnet 1999 [152]	France	PCR, IHC	51/100 50%	0/30 0%	0.001
Fina 2001 [153]	Algeria Europe	PCR, ISH, microdissection	162/509 32%	0/10 0%	0.001
Grinstein 2002 [154]	United States	PCR, IHC	14/33 42%	3/26 12%	0.039
Preciado 2005 [155]	Argentina	PCR, IHC	24/69 35%	0/17 0%	0.001
Fawzy 2008 [156]	Egypt	PCR, IHC	10/40 25%	0/20 0%	0.001
Joshi 2009 [157]	India	IHC	28/51 55%	0/30 0%	0.001
Lorenzetti 2010 [158]	Argentina	PCR, ISH, IHC	22/71 31%	0/48 0%	0.001
Kadivar 2011 [159]	Iran	PCR, IHC	0/100 0%	0/42 0%	
Mazouni 2011 [160]	France	PCR microdissection	65/196 33%	1/15 7%	0.001
Hachana 2011 [161]	Tunisia	PCR, IHC	33/90	0/123	0.001s
Glenn 2012 [37]	Australia	PCR, in situ PCR, IHC	34/50 68%	14/40 35%	0.011s
Zekri 2012 [162]	Iraq	PCR, IHC, ISH	32/90 35%	0/20 0%	0.001s
Khabaz 2013 [163]	Jordan	PCR, IHC	24/92 26%	3/49 6%	0.001s
Yahia 2014 [164]	Sudan	PCR, ISH	49/92 53%	12/50 24%	0.001s
Mohammadizadeh 2014 [165]	Iran	PCR, IHC	6/74 8%	0/80 0%	0.001s
Richardson 2015 [166]	New Zealand	PCR	24/70 34%	9/70 13%	0.253 ns
Ahmed 2016 [167]	Egypt	IHC	11/10,710%	0/107 0%	0.001s
El Naby 2017 [168]	Egypt	PCR, IHC	10/42 24%	6/42 14%	0.689 ns
Fessahaye 2017 [169]	Eritrea	PCR, ISH, IHC	40/144 28%	4/33 12%	0.003s
Pai 2018 [170]	India	ISH	25/83 30%	0/7 0%	0.001s
Al Hamad 2020 [45]	Jordan	ISH	24/100 24%	0/20 0%	0.007s
Alinezhad 2021 [171]	Iran	PCR	9/80 11.2%	0/80 0%	0.009s
Nagi 2021 [138]	Lebanon	PCR	41/102 40%	0/14 0%	0.001s
Zhang W. 2022 [172]	China	PCR	54/140 41%	0/25 0%	0.001s
Khasawneh 2024 [141]	Jordan	PCR	18/110 16%	1/30 3%	0.001s

PCR = polymerase chain reaction, IHC = immunohistochemistry, ISH = in situ hybridisation, ns = not significant at 0.05 level

Epstein–Barr virus gene sequences have been identified in benign breast tissues 1 to 11 years prior to the development of EBV-positive breast cancer [145]. This is an important causal criteria.

In economically developed countries EBV associated infectious mononucleosis occurs most commonly among teenagers. This is in contrast to developing countries where EBV infections mainly occur in early childhood. EBV infections in teenagers and young adults is associated with Hodgkins lymphomas. There is a strong correlation between EBV associated Hodgkins lymphoma and breast cancer [173]. Epstein–Barr virus is mostly transmitted from person to person via saliva. EBV has been found in 61% of blood samples from healthy donors, which may explain its transmission through the body [174].

#### Oncogenic mechanisms

EBV infections predispose human breast epithelial cells to malignant transformation [175]. EBV EBNA-1 has been associated with BRCA- 1 gene defects which in turn is associated with breast cancer [176]. The precise oncogenic mechanisms for EBV are not known.

### Conclusions

The evidence for a role of EBV in breast cancer while consistent, needs to be further developed.

## Bovine leukemia virus

Bovine leukemia virus is an oncogenic retrovirus capable of integrating into a host’s DNA causing a lifetime infection. Janice Miller and colleagues of the US were the first to identify virus like particles in cattle lymphosarcoma in 1969 [177]. These particles became known as bovine leukemia virus. Only a small proportion of infected animals develop cancer – most of which are lymphomas.

BLV infects cattle in the Americas, some parts of Europe and Asia plus the Middle East. Breast cancer is more prevalent in red meat eating and cow’s milk consuming populations as compared to those with a high prevalence of lactose intolerance such as China [178]. In cattle BLV is mainly located in lymphocytes and mammary epithelial cells which can exfoliate into milk.

In 2003 Gertrude Buehring and her colleagues at the University of California at Berkeley were able to identify BLV antibodies in human blood serum [179]. Later, Buehring and her colleagues, identified BLV in 44% of US breast cancers [180]. BLV has since been identified in breast cancers in women from Australia, Argentina, Columbia, Brazil, Iran and Pakistan but not in Europe, China or Japan. The nucleotide sequences of the BLV *env* gene are 97.8 to 99.7% the same in both human breast tissues and cattle blood [181]. This indicates that BLV is probably a zoonotic infection.

Using whole genome sequencing Gillet and Willems did not identify BLV DNA in any of 51 human breast cancer sequences based on the US National Center for Biotechnology and Information [182]. This is contrary to the outcomes based on serology and PCR. A possible explanation suggested by Vinner et al. 2015 is the extremely low BLV load in human breast cancer [183]. Amato et al. using PCR did not identify BLV in US breast cancers [184]. The reason is not known.

Based on 9 case control studies, the prevalence of BLV was 334 (40%) of 826 breast cancers.

compared to 215 (24.0%) of 898 normal and benign breast controls [8]. With two exceptions the prevalence of BLV is significantly higher in breast cancers as compared to controls. In a meta-analysis of case control studies BLV was associated with an odds ratio of 2.6 increased risk of breast cancer [8]. These case control studies are shown in Table 7.

**Table 7:** Identification of bovine leukemia virus in human breast cancer (case control studies)

Study	Location	BLV positive breast cancer	BLV positive normal benign breast	Significance
Giovanna 2013 [185]	Columbia	19/53 36%	24/53 45%	0.682 ns
Buehring 2015 [186]	US	67/114 59%	30/104 29%	0.001s
Zhang R 2016 [187]	China	0/91 0%	0/100 0%	
Buehring 2017 [188]	Australia	40/50 80%	19/46 41%	0.001s
Baltzell 2017 [189]	US	35/61 57%	20/103 20%	0.059 ns
Khalilian 2019 [190]	Iran	57/172 30%	5/28 8%	0.001s
Schwingel 2019 [191]	Brazil	22/72 30.5%	10/72 13.9%	0.017s
Delamelina 2020 [192]	Brazil	47/49 96%	23/39 59%	0.001s
Canova 2021 [181]	Brazil	51/59 86%		
Khan 2022 [193]	Pakistan	728 /2710 27%	10/80 13%	0.005s
Yamanaka 2022 [194]	Japan	0/23 0%		
Amato 2023 [184]	US	0/56 0%		

ns = not significant at 0.05 level

Of special interest is a study of BLV in human breast cancers in south eastern Brazil where the people traditionally ingest raw (unpasteurized) milk and cheese. BLV was present in 90% of the dairy cattle and 96% of human breast cancer cases [192].

In a study of Australian women with breast cancer, BLV was identified in benign breast tissue 3–10 years before BLV positive malignancy was diagnosed [188]. This observation is in accord with the causal criteria of a prior infection before the development of the same pathogen related cancer. In this same study BLV was identified in high proportions in both breast and benign controls (80% and 41% respectively). As only a small proportion of BLV infected animals develop cancer it is possible that only a small proportion of BLV infections in humans also progress to breast cancer.

BLV has not been identified based on PCR in breast cancers in several studies including Japan [194] and the US [184]. Canova et al. (2021) suggest that these conflicting results might be related to differing PCR methods [181]. It is possible that the viral DNA sequences targeted by the PCR may not have been present in the genomes that were analysed. Partial genome deletions following integration into the host cells are common and can be an important mechanism to avoid the host immune response. Such deletions have been observed in studies of BLV-related primate T lymphotropic virus type 1 (PTLV-1), including deletions in the *gag* region, followed by deletions of the *pol* and *env* genes. In contrast, the *tax* and *LTR* regions were the less frequently deleted genes [195].

### Transmission

BLV has been identified in up to 49% of fresh milk and raw beef available for human consumption [196, 197]. Further, BLV RNA has been identified in the air and on surfaces at dairy workplaces, which may be a source of occupational infection [198].

### Oncogenic mechanisms

Due to the economic importance of BLV in the cattle industry there have been detailed investigations into its oncogenic mechanisms. BLV encodes the regulatory protein Tax. It is the key protein involved in viral replication. In animals BLV is a three stage process. (i) BLV infection of cells, (ii) immortalisation of cells by the influence of Tax proteins and (iii) malignant transformation following p53 and other mutations [199]. Only a small proportion of infected animals develop cancer – most of which are lymphomas. Although it is likely that the oncogenic mechanisms of BLV are similar in humans, there is no evidence available.

### Conclusion

It is likely that BLV has a causal role in some human breast cancers but additional evidence is required before any conclusions can be made.

## Inter-relationship between MMTV, HPV, EBV and BLV in human breast cancer

MMTV, HPV and EBV have been identified in the same Australian breast cancers [37].

In addition these multiple viruses have been identified in benign breast specimens 10 years before the development of the same multiple virus associated breast cancers in the same women [145]. Co-infection of high-risk HPV and EBV, has been observed in Lebanese and other breast cancers [138]. As outlined above HPV appears to influence the oncogenicity of MMTV via its influence on APOBEC enzymes [95]. The oncogenic influences between HPV and EBV are not known.

## Discussion and conclusions

### Mouse mammary tumour virus

The evidence that MMTV has a causal role in human breast cancer is increasingly comprehensive. The oncogenic influences of MMTV in human breast cancer appears to be almost identical to MMTV in mice. In Western women, the prevalence of MMTV in breast cancers is approximately 30 to 40%. In China, Korea and Vietnam, the prevalence of MMTV is 10 to 20%. Overall the odds ratio between MMTV in human breast cancer and normal and benign breast tissues is very high at 13·40. There appears to be an association between MMTV positive breast cancer and the location of MMTV infected Mus *domesticus* mice. The life cycle of MMTV in humans is similar that of mice although the means of transmission probably differs. In humans the most likely means of transmission is via sputum whereas in mice transmission is via mouse milk from infected mother to pup.

While it is likely that MMTV has a causal role in human breast cancer the development of additional evidence is of advantage. In addition to the above evidence there is a need to replicate the Graff et al. studies on mice conducted in 1949 in which MMTV particles isolated from mouse milk were injected into the perineum of healthy mice. Approximately half of these mice developed breast cancers [11]. It should be possible to isolate MMTV particles from human breast cancers and inject them into experimental mice.

#### Conclusion

The evidence meets the extended Hill causal criteria. A causal role for MMTV-like viruses in human breast cancer is probable.

### Human papilloma viruses

High risk for cancer HPVs have been consistently identified and are significantly more prevalent in breast cancers than normal and benign breast cancers. There have been 46 case control studies in 20 countries in which high risk for cancer HPVs have been identified [6]. The prevalence of high risk for cancer HPVs was 1335 (31%) of breast cancers as compared to 163 (9%) of 1838 normal and benign breast tissue controls (*p* = 0.001). Overall the odds ratio between high risk HPVs in breast cancer and normal and benign breast tissues is 5.56. While sexual intercourse is accepted as the main means of HPV transmission, there is evidence that HPVs may be transmitted via saliva and blood and from the cervix to the breast by circulating extra cellular vesicles – also known as exosomes.

The causal mechanisms for HPV related breast cancer appear to differ from cervical cancer. While HPV proteins E6 and E7 probably have a causal role as demonstrated by the presence of HPV related koilocytes (characteristic cells used as the basis of Pap smears) in breast cancer, HPVs in breast cancer appear to have two additional causal mechanisms by (i) influencing APOBEC mechanisms (APOBEC enzymes offer antiviral protection) and (ii) combining with EBV. In conclusion it is likely that HPVs have a causal role in breast cancer.

#### Conclusion

In conclusion it is likely that HPVs have a causal role in breast cancer.

### Epstein Barr virus

EBV has been consistently identified and is significantly more prevalent in breast cancers than normal and benign breast cancers. The prevalence of EBV was 844 (31%) of 2754 breast cancers as compared to 53 (5%) of 1061 normal and benign breast tissue controls (*p* = 0.001). Overall the odds ratio between EBV in breast cancer and normal and benign breast tissues is 4.43. The underlying mechanisms for a role of EBV in breast cancer is not clear.

#### Conclusion

The evidence for a role of EBV in breast cancer while consistent, needs to be further developed.

### Bovine leukemia virus

Based on 9 case control studies, the prevalence of BLV was 334 (40%) of 826 breast cancers compared to 215 (24.0%) of 898 normal and benign breast controls. Overall the odds ratio between BLV in breast cancer and normal and benign breast tissues is 2.26. The associations between the presence of BLV in fresh meat and milk and increased prevalence of BLV positive breast cancer is suggestive of a causal role for BLV.

#### Conclusion

It is likely that BLV has a causal role in some human breast cancers however additional evidence is required before any conclusions can be made.

### Susceptibility to virus associated breast cancer

Only a small proportion of women exposed to the established risk factors for breast cancer – early age menarche, late age menopause, late age first pregnancy, excess weight, genetics, develop breast cancer. There is no available evidence relevant to viruses and breast cancer. The most plausible reason why some women exposed to these risk factors, including virus infections, develop breast cancer is genetic susceptibility [200, 201]. This susceptibility may be familial or sporadic.

### Prevention of viral induced breast cancer

Effective vaccines against HPV infections are widely available for both girls and boys [202]. Recently a successful vaccine against BLV in cattle has been developed [203]. This is a crucial development. In future the use of culling (killing) BLV infected cattle should no longer be necessary.

Vaccines against MMTV and EBV for use in humans are not available and require urgent development. Using traditional methods vaccines have been successful in the prevention of MMTV associated breast cancer in mice [204].

## Data Availability

All the relevant data are within the paper.
